# Retraction Note: Analysis of fractured soft rock characteristics in fault rupture zones and laneway shoring

**DOI:** 10.1038/s41598-023-46687-8

**Published:** 2023-11-07

**Authors:** Xiangdong Zhang, Yu Zhang, Jianjun Yang, Lijuan Su, Wenliang Li, Jie Geng, Zong Li, Xuefeng Zhang, E. Fei

**Affiliations:** 1 School of Civil Engineering, College of Civil Engineering, Liaoning Technical University, Fuxin, 123000 Liaoning China; 2 China Northeast Architecture Design and Research Institute Co., LTD., Shenyang, 110000 Liaoning China

Retraction of: *Scientific Reports* 10.1038/s41598-023-43475-2, published online 26 September 2023

The Authors have retracted this Article. After the publication of the Article, the Authors discovered that their TAW-2000 rock testing machine had developed a malfunction. The machine was used in most of the experiments and the Authors cannot be certain at which point the machine failed. The Authors therefore no longer have confidence in the data.

All Authors agree to this retraction.

